# Modified Listeriam in Ovariotomy, with a Report of Five Recent Operations

**Published:** 1883-04

**Authors:** Edward W. Jenks

**Affiliations:** 170 State St., Chicago, Illinois


### Selections.

---

### Modified Listerism in Ovariotomy, with a Report of Five Recent Operations. By Edward W. Jenks, M.D., Chicago, Illinois.

At the meeting of the Illinois State Medical Society, held in May last, I read a paper in which were embodied my own views regarding Listerism in ovariotomy.

Since the above mentioned paper was written, I have had opportunities of carrying out in practice in several cases, the views I expressed, but as the transactions of the society * are seen only by a limited number, I offer no apology for repeating in substance some of the expressions there made use of.

The readers of this journal are all, doubtless, familiar with the discussions which have taken place in various parts of the civilized world in regard to Listerism in ovariotomy.

It is well known that Keith has ceased to rely on it as he formerly did—having virtually discarded it. At the Clinical Society of London, Lister said but a few months ago, in commenting on a death from carbolic acid, that it is too powerful to safely apply in delicate subjects. This topic was discussed at the last International Medical Congress held in London, where it was expected that Lister would speak in its defense, but he remained silent. Keith there stated that he had discontinued Listerism in his ovariotomy operations. The subject was also discussed by many distinguished operators, including some of our own countrymen. In this country there is an unsettled belief regarding its utility,

---

* The trancactions for 1882 are about ready for distribution. I have this week (Nov. 25, 1882), in reading the proof of my paper, added a foot-note referring to these five cases of ovariotomy.

and while there are some gynæcologists who still adhere to pure Listerism in their ovariotomies, there can be no question that the number is less than it was two years ago.

I am convinced, as stated in the paper first referred to, that I have seen two fatal cases of ovariotomy in consequence of the use of carbolic acid spray, and hence am led to believe that a carbolized solution or spray of sufficient strength to destroy bacteria, is an unsafe agent to come in contact with the peritoneum.*

While it is apparent that the adherents of pure Listerism in ovariotomy are gradually diminishing in number, there is no mistaking the fact that since Lister promulgated his theories regarding antiseptics, and carried them into practice, the mortality in ovariotomy has been greatly diminished. While the percentage of recoveries following ovariotomy has been greater since Lister introduced his system, there is one thing pertaining to it that is not so certain, namely: that it has been wholly due to carbolic acid. The profession has been taught by means of Listerism that one of the greatest aids to recovery from surgical operations is perfect cleanliness. All honor, then, I say, to the name of Lister, to whom not the profession only is indebted, but the whole world. Notwithstanding many distinguished surgeons have expressed themselves in strong language against pure Listerism in ovariotomy, all seem to agree that the originator is entitled to our lasting gratitude.

Without quoting from, or even attempting to name the long list of those who have thus expressed themselves, I will merely add for myself that instead of wholly renouncing Listerism in ovariotomy, I am a warm advocate of what may be termed a modified Listerism. The modified form is characterized chiefly by careful attention to perfect cleanliness. The spray can be used in the room prior to the operation, but is not deemed of paramount importance during the exposure of the peritoneum to the atmosphere. Carbolic acid on every instrument, suture, dressing and appliance used, is of less importance than that they should be perfectly clean.

* I am assured by those who have experimented in the matter, that a ten per cent. solution of carbolic acid is the weakest that can be relied on to destroy bacteria, and such a solution will be disastrous in its effect if applied to the peritoneum.

In my own practice, it is possible that I have gone further than many others in regard to carbolic acid, as I consider it such an irritant that I make it a rule to carefully exclude it from contact with the peritoneum. The fluid for the atomizer, if carbolized in the least, I do not have contain over two per cent. of the acid.

The atomizer is, without doubt, of great benefit, if for no other purpose than to render the atmosphere of the operating room in a more favorable condition for exposing the peritoneum, and for this purpose pure water or a two per cent. solution of carbolic acid will suffice.

I can better illustrate the modified Listerism I have used in my ovariotomies, by referring to recent operations—five in number. Of these five, one was unsuccessful. At least three of these may be classed as complicated, and hence, serious cases. For the sake of brevity many matters not pertinent to the subject will be omitted, but there will be given an account in outline of the steps of each operation, and afterwards a summary of the treatment, etc., used in the whole number.

CASE I.—Mrs. M., æt. 36, of N., Mich., when she entered the hospital the last of April, stated that she had been tapped twice, but was unable to say how much fluid had been drawn off at either time. She was then suffering considerable pain through the abdomen, with daily rigors and fever, which I thought might be of malarial character.

I aspirated the tumor for the purpose of examining its contents microscopically. The fluid was found, by my friend Dr. Mercer, to contain fully fifty per cent. of pus.

This being the second suppurating cyst I had ever met with, and having been led by past experience to believe that delay would not lessen the constitutional symptoms, I decided to operate as soon as possible.

Preparatory treatment for a week consisted of daily warm baths followed by inunction and full doses of quinia and iron. The night before I operated, her temperature was 102°.

The morning of the operation ten grains of quinine were administered per rectum. The room was thoroughly filled by the carbolized vapor (two per cent.) by means of anatomizer. Every attention was paid to cleanliness. The ligatures were not carbolized, but clean and well waxed. The spray, during the operation, was not directed toward the patient's abdomen, but, more from oversight than any other reason, was in operation in one corner of the room. The anterior portion of the tumor was so firmly adherent that to detach it was a long and tedious process; there were but few other adhesions and they were easily detached. The pedicle was secured by means of waxed silk, cut short and dropped back. The abdomen was not closed until there was a certainty of the cavity having been thoroughly sponged out and that there were no bleeding points. The tumor was multilocular and was estimated to weigh over thirty pounds. The wound was closed with silver sutures and dressed antiseptically.

This patient made a rapid recovery. Whenever any rise in temperature occurred, from ten to fifteen grains of quinine were administered, either hypodermically or per rectum. At no period of her convalescence did she experience so much discomfort as during the night previous to the operation.

CASE II.—Mrs. O., æt. 50 years, was sent to me by Dr. Muth, of Sheboygan, Wis. This patient was put on the same preparatory treatment as in the preceding case, and on the day of the operation, April 22d, 1882, ten grains of quinine were administered per rectum. The operation was made in the presence of about twenty-five physicians. The room was carbolized by the atomizer, the solution, however, containing only two per cent. of carbolic acid.

There were no particular complications, except adhesions to the omentum, requiring ligation and cutting away of one-fourth of it. No carbolic acid was allowed to enter the peritoneal cavity, nor were the ligatures carbolized, but perfect cleanliness was aimed at. The assisstants were each required to wash their hands thoroughly with soap and water by means of a nail brush. The abdomen of the patient was also washed with soap and water, and wiped dry prior to making an incision. The pedicle was ligated with silk, both ends cut short and dropped into the abdomen. Especial pains was taken in sponging out the cavity, that not a drop of blood should be left.

The tumor weighed thirty-nine pounds. Silver wire was used to close the abdominal incision, over which were placed the usual antiseptic dressings, held in place by a flannel bandage.

The after treatment consisted of sufficient morphia to control pain. The temperature was kept down by quinia. During the first week all nourishment was given per rectum.

CASE III. Mrs. B., æt. 29, of H., Mo., was referred to me by Dr. West, now a resident of Monroe, Mich. She had been seen by a number of physicians of repute, and by some of them the case was not considered a favorable one for operation, as the tumor was believed to be extensively adherent.

The usual treatment, as in preceding cases, was prescribed for a week prior to operating. The patient was placed in a private house, temporarily fitted up as a private hospital. I operated May 18th, assisted by Drs. West, Newman, of Chicago, Mitchell, of Iowa, McLean, of Tilsonburg, Ontario, and others. The condition of every instrument and appliance that might possibly be required was made as clean as possible. The spray, with a two per cent. solution of carbolic acid, was used in the room for an hour previous to operation, so that the atmosphere of the room was very humid and of high temperature.

The adhesions were extensive and in every direction, the tumor being adherent to the abdominal walls over nearly its entire anterior surface, to the uterus, bladder, intestines and liver.

The detachment from the liver required the most careful manipulation, but fortunately it was done without injury to that viscus. The pedicle was ligated and other procedures in the operation done as already described. Several bleeding points were secured by silk ligatures and oozing surfaces treated by hot water; but one bleeding locality could not be secured by either of these methods; that was from the abdominal wall near the liver, where the tumor had been firmly adherent. This bleeding surface, of an area as large as the palm of the hand, to which ligatures could not be applied, and hot water would not check, I finally controlled by passing a needle armed with a strong silk ligature from the outside into the abdominal cavity across the oozing surface described, then bringing the needle outside, and by means of a ligature, tying in a fold this portion of the abdominal wall. The bleeding was effectually stopped, and at the end of twelve hours the ligature was removed without doing harm, but good, instead. This patient made a speedy recovery. The carefully kept record shows that several times when there was a rise in the temperature, quinia in ten-grain doses caused it to descend to the normal point.

CASE IV.—Mrs. S., æt. 38, of G., Ill. This patient was not seen by me until I was called to her residence by telegraph to perform ovariotomy, June 25th. The patient seemed in good condition, and everything looked favorable for her recovery. The usual steps in the operation, as already described, were taken, except that no spray was used. I have never taken more pains to see that everything used about the patient was in a state of perfect cleanliness. The instruments were washed in carbolized water, and after the operation, antiseptic dressings were applied. The tumor weighed about fourteen pounds, and had no adhesions except some slight ones in front.

The weather was exceedingly hot, and at the moment when I was about to secure the pedicle there came one of the most terrific thunder storms I have ever witnessed. The room became so dark that lamps were necessary to even see the patient, and in such a dim light as can be better imagined than described, the operation was completed.

She rallied well, and when I left her, eighteen hours after the operation, seemed in a fair way to recover, yet I could but feel apprehensive as to the result, unless there should be a change in the weather.

Unfortunately the change did not come until too late to be of service in this case. The thermometer ranging from 90° to 95° F., with frequent thunder storms continued for several days. This was at the time when all through the west there were cyclones, tornadoes, and thunder storms of daily occurrence. The patient died the third day after the operation, and from all I can learn, I can but believe that the result was attributable to the condition of the atmosphere.

CASE V.—Mrs. H., æt. 52, of Oconto, Wis., first consulted me March 2, 1882, bringing a letter from her physician, Dr. O'Keef, of the same place.

The tumor was then quite small and the patient had such a dread of an operation that I advised her to return home, at the same time saying that in all probability ovariotomy would be necessary some time. After a time I was telegraphed for, to come to her residence and remove the tumor. On my arrival (May 27th) I found her in a very feeble condition, with the abdomen greatly distended. She was so feeble that an examination caused syncope, and hence the operation of extirpation of the tumor was indefinitely postponed. But there was so much œdema of the lower limbs, and dyspnœa, that it was decided to risk tapping the cyst. I had no aspirator, nor could one be obtained before my return home. I accordingly tapped with a small trocar and drew off twenty-six pounds of fluid. This was much better borne than we expected. She began at once to improve in strength, and in a few days was about the house.

Six weeks later, Dr. O'K. again tapped the tumor, and drew off nearly as much fluid as in the first instance. It was not believed by either Dr. O'K. or myself that the operation of extirpation would ever be performed, and so what was done was considered as merely palliative. I was accordingly greatly surprised to receive a message to come to Oconto and remove the tumor, which was accomplished August 16th. The weather being cool, the room was heated with a stove and then filled with warm vapor by means of the atomizer and so kept during the operation ; this I consider fortunate in view of the fact that the peritoneum was exposed to the air an unusually long time.

Believing the bladder to be adherent, I made the exploratory incision a little below the umbilicus, and even then barely escaped wounding the bladder, as its adhesion to the tumor had caused it to be thus elevated.

The adhesions in this case surpassed any I ever encountered before in number and extent. The tumor was adherent to the abdominal wall, the bladder, uterus, mesentery, intestines* and omentum.

The detachment of adhesions required a long time, the most difficult ones being the intestinal and mesenteric. Owing to so many adhesions there was presistent bleeding from many points. To these, ligatures were applied in greater number than I ever used or witnessed before.

---

*The adhesions to the intestines were quite similar to a case described in the *British Medical Journal*, Oct. 28, 1882.

There were upwards of thirty, including those used on the pedicle, of which none were carbolized, but all had been in boiling water, speedily dried and then waxed. We ceased counting the ligatures after thirty had been used.

The tumor weight thirty-five pounds. After closure of the abdominal incisions the usual antiseptic dressings were applied. I remained with the patient the day following the operation, after which she was left in charge of Dr. O'Keef, of whom I cannot speak in excessive praise.*

This seemed to be one of those cases where a drainage tube was demanded owing to the great number of adhesions, yet as every point seemed to be secure, and the cavity so thoroughly wiped out, it was thought best not to insert one.

The patient was fed and stimulated almost wholly by rectum for fourteen days. I felt greatly encouraged about her recovery, owing to the fact that twelve hours after the operation there was a passage of intestinal flatus. This is an invaluable prognostic sign following the operation.

August 28, I received a telegram from Dr. O'K. stating that the patient showed signs of septicæmia. I replied that if the symptoms did not very soon yield to quinine and stimulants, he had better open the wound and wash out the abdominal cavity. He prepared to do so, but soon after the treatment was instituted she began to improve and without further drawback gradually recovered.

Successful ovariotomies are now so common that the reports of ordinary cases possess no particular interest for the profession, hence I have aimed in the foregoing brief record to mention only what may be termed the most important points. I have stated that I avoided the admission of carbolic acid to the peritoneal cavity, and yet had a carbolized spray in the room. It is true that in this way some of the carbolic acid may enter the peritoneal cavity, but it is in so small an amount as to differ altogether from the playing of a spray directly towards the abdomen of the patient.

---

*It is quite important that I should also refer to the preparatory treatment in this case. This had been carried out by Dr. O'Keef in accordance with the plan recommended in this paper for several weeks preceding the operation; consequently she was in much better condition than at my previous visit.

Of the foregoing cases all were treated antiseptically, yet not after the method of Lister. Although the spray was used its chief advantage was in rendering the atmosphere humid, as, being only a two per cent. solution, it could not destroy bacteria.

When I have used the ordinary solution by the atomizer, my own hands have been rendered almost useless by the continuous action on them of the carbolic acid, and the peritoneum of the patient whitened by it. It looks reasonable, then, that so delicate a membrane as the peritoneum must be seriously damaged by carbolic acid when a five per cent. solution produces such effects. There may be advantages, even in a weak solution, of which we have no knowledge, and therefore regret that I did not use it in the fatal case, yet do not believe in this instance it would have caused any other result.

I can see no objection in using a five per cent. solution in the room prior to an operation. It may even be well to have a stronger solution atomized on the persons of the assistants and spectators, or even the surgeon himself if there is reason for thinking they may be media for conveying infection ; but I must continue to enter my protest against full Listerism on account of the poisonous effects of a strong solution of carbolic acid on peritoneum, and I believe that while we may carbolize the room and spectators prior to operating, we should carefully exclude carbolic acid from the peritoneum, whether by spray, instrument, sutures, sponges, or the hands. Cleanliness is of greater importance than carbolic acid.

I had written the foregoing, when it occurred to my mind to refer to the paper written by my friend, Dr. Englemann, of St. Louis, entitled "Difficulties of Ovariotomy," and published in the *American Journal of Medical Science*, for April, 1882, which I read at the time of its appearance, but had not since seen. The paper is an admirable and instructive one, and I heartily endorse its teachings. It concludes with some "points" which he urges on his readers, from which I select the following as pertinent to my subject: "Avoid routine Listerism, and especially the carbolic acid spray over the hands of the operator and into the abdominal cavity." "Cleanliness, not carbolic acid, is necessary." "Keep sponges clean and warm, but not carbolized." "Ligatures, sutures and instruments should be clean but not carbolized."

In connection with the subject of Listerism, there is another material besides carbolic acid for which in the outset great things were claimed, viz.: catgut, as a material for ligatures and sutures. My own views regarding this material in obstetrical and gynecological surgery have been heretofore expressed in several papers. A translation of one which was written for a foreign journal* was published in the *News* in 1878. In that paper I gave reasons sustained by facts showing that catgut possessed no advantages in the peritoneal cavity over silk, but on the contrary had been in many instances a positive damage. While catgut may be serviceable in general surgery as in stumps after amputation, and in other parts of the body, it should not be used in ovariotomy, as the tying of a knot securely is not only difficult but the material, being apt to stretch on account of the moisture of the peritoneal cavity, is rendered worthless. Further I have observed in post mortem examinations, where catgut had been previously used in the peritoneal cavity, that it is not less innocuous than silk. I am therefore led to the firm belief that there is no better material for ligatures or sutures than clean non-carbolized silk.

There are many other matters of interest relating to the preparatory and after-treatment of ovariotomy patients, the consideration of which must be omitted from this paper as they do not strictly come within its scope. There are some points, however, bearing upon the prophylaxis of septicæmia (doubtless the most frequent cause of death following the operation) to which brief allusions will be made. I heard not long since a gentleman of repute as a surgeon gynecologist say in quite a boastful manner, "I never give a dose of medicine to my patients until symptoms clearly demand a remedy—nothing to prevent disease—not even a dose of quinine in my ovariotomy cases to prevent septicæmia or for any other reason. When there are symptoms of septicæmia, then I give it." Is there not danger of carrying our contempt for drugs too far in these days when "dosing" is so vigorously assailed? As I consider the prophylaxis of disease one of the most important functions of the medical pro-

* *Archives de Tocologie*, 1878, *p.* 108 "*Sur les sutures de l'utérus das l'opération Césarienne, par Dr. E. W. Jenks.*"

fession, I differ most decidedly from my friend just quoted. The majority of ovariotomists are aiming to prevent septicæmia when they prescribe baths, inunctions, tonics, and other remedies in preparatory treatment. As quinia is considered the remedy *par excellence* in the treatment of septicæmia, why not administer it as a preventive? In all of my ovariotomies for the past three years, when I have had control of the preparatory treatment, I have given quinia freely for the week preceding the operation, and ten or fifteen grains within an hour of beginning it.

Further, when after the operation there has been a rise of temperature, I have administered quinia either hypodermically or per rectum, with markedly good results, being guided in the amount and frequency by its effect. For hypodermic use, the bisulphate is preferable, on account of its ready solubility in water, and consequently lesser liability of causing abcesses.

To attain success in the greatest number of cases (without taking into consideration the selection of patients), operations should not be made in general hospitals, nor at long distances from the surgeon's residence, as he then is often of necessity obliged to intrust patients to the care of inexperienced and untrained attendants and nurses.

In support of the foregoing: First, sufficient evidence is found in the statement recently published in the medical journals to the effect that the first successful ovariotomy in Bellevue Hospital, New York, is of a very recent date—only since the completion of a new pavillion where it was performed. Second, the reader is referred to statistics and remarks relating to the late Prof. Peaslie's ovariotomies, published in the *American Journal of Obstetrics* for October, 1882, where it is shown that the large percentage of fatal cases were greatly owing to operations made far from his own home and leaving the patient to the care of others thus really having nothing personally to do with either preparatory or after-treatment. The best places for ovariotomies are well-regulated private hospitals, with well-trained nurses and attendants, or private houses for the time being practically converted into private hospitals, with the same kind of nurses and attendants. The advantages of such arrangements cannot but be obvious, and certainly require no lengthy argument in their behalf.

170 State St., Chicago, Ill.

